# Smaller birth size may be associated with lower physical activity in adult women

**DOI:** 10.1038/s41598-025-14620-w

**Published:** 2025-08-20

**Authors:** Kinga Słojewska, Andrzej Galbarczyk, Magdalena Klimek, Magdalena Mijas, Anna Tubek-Krokosz, Karolina Krzych-Miłkowska, Monika Ścibor, Grazyna Jasienska

**Affiliations:** 1 Department of Environmental Health, Faculty of Health Sciences, Jagiellonian University Medical College, Krakow, Poland; 2 Doctoral School of Medical and Health Sciences, Jagiellonian University Medical College, Krakow, Poland; 3 Department of Human Behavior, Ecology and Culture, Max Planck Institute for Evolutionary Anthropology, Leipzig, Germany; 4 Department of Anthropology, Baylor University, Waco, USA

**Keywords:** Birth size, Developmental conditions, DOHaD, Energy expenditure, Physical activity, Ponderal index, Evolutionary developmental biology, Health care, Behavioural genetics, Evolutionary biology

## Abstract

Conditions during fetal development are crucial for a long-term health. Individuals with small size at birth are suggested to have energy-thrifty physiology, a tendency to conserve energy due to adaptations to undernutrition during early development. However, energy also could be saved by having low physical activity. We hypothesize that individuals born smaller are less physically active compared to those born larger. Data were collected from 136 healthy, urban women (mean age 26.6, SD 4.24) over 3 consecutive menstrual cycles. Cycle 1 involved usual physical activity, while in cycles 2 and 3 women were instructed to perform at least 180 min/week of moderate to vigorous activity. Birth weight and length were obtained from personal medical records, and physical activity was monitored using a Fitbit wristband accelerometers. For each woman, ponderal index (indicator of leanness at birth) was calculated. Smaller size at birth predicted lower total energy expenditure during adulthood. The ponderal index was positively associated with duration of vigorous physical activity across all cycles (cycle 1: *p* < 0.001; cycle 2: *p* = 0.011; cycle 3: *p* = 0.004), while the birth weight was positively related to total energy expenditure (cycle 1: *p* = 0.014; cycle 2: *p* = 0.008; cycle 3: *p* = 0.016). Fetal developmental conditions might be associated with physical activity levels in later life. Women born smaller have lower total energy expenditure and are less likely to engage in vigorous activity. These findings underscore the role of early life factors in shaping health-related behaviors and suggest that individuals born with smaller size may need additional support or tailored interventions to meet the recommended levels of physical activity.

## Introduction

Physical activity is crucial for human health, e.g. by reducing the risk of non-communicable diseases, especially cardiovascular diseases, diabetes and cancer^1,2^. Physical activity has been indispensable for survival in hunting and gathering populations, thus our evolutionary ancestors had much higher levels of activity than most contemporary people^3–5^. It is estimated that about 40% of members of modern societies worldwide have insufficient levels of physical activity^6^. This problem has been addressed by numerous worldwide guidelines (e.g. issued by World Health Organization) and public health initiatives aimed to increase the amount of physical exercise^7^. Evolutionary approach helps to understand why, in general, organisms have low leisure-time physical activity^8,9^. Expending energy on activities that are not necessary for survival, or more generally, do not increase evolutionary fitness, may lead to negative energy balance, which has detrimental effect on reproduction^10^ and health^11^. Thus, it is understandable that mechanisms, mostly behavioral, limiting leisure-time physical activity would be promoted by natural selection. These energy saving mechanisms are especially important for organisms developing in environments that are poor in energy, and particularly, when poor conditions are persisting for a longer time, as in case of individuals that receive a signal about energy restrictions during their prenatal stage of life^12,13^.

Understanding these evolutionarily ingrained behavioral patterns has important public health implications. It underscores the need for early identification of individuals who may be predisposed to low physical activity and the necessity for the development of targeted interventions. Since physical inactivity is a modifiable risk factor for chronic diseases, addressing it is critical for health promotion.

Developmental conditions, especially during fetal life, have profound influence on physiology and health in later life^14–21^. Poor developmental conditions might lead to changes in the organism (e.g. structural, functional or behavioral) reflecting developmentally plastic responses to environmental signals^22^. Individuals developing in energetically poor conditions often not only have small size at birth but also possess physiology and metabolism that differ from those individuals that did not experience energy restrictions^23^. It is postulated that small size and accompanied physiological changes are adjustments for anticipated poor access to energy during post-natal life^20,21^. In this framework, poor fetal conditions serve as predictive signals of potentially energy and nutrient-scarce conditions during childhood and adulthood.

Most studies on the role of developmental “programming” investigated changes in physiology and health, documenting problems with glucose metabolism, hypertension, weight gain, ovarian hormones, and higher risk of diseases, especially cardiovascular diseases and diabetes, in individuals with small size at birth^24–32^. A low birth weight has been consistently associated with an increased risk of metabolic disorders, including insulin resistance, type 2 diabetes, and cardiovascular disease, later in life^12,33–35^.The physiological changes that increase risks of these health problems may be a consequence of physiological adjustments that serve to save energy. However, if poor conditions during fetal development indeed “program” individual to be more energetically thrifty, such programming might not be restricted only to physiological mechanisms but could also include behavioral ones. Individuals would function better in energy restricted environment if they reduced their physical activity level and thus their energy expenditure.

Not many studies have examined developmental programming of behaviors such as physical activity^36^. In animal models, undernutrition in utero led to decreased adult physical activity^37,38^. Similarly, human studies have also shown that lower birth weight may be associated with decreased muscle strength^17,39,40^ muscular endurance^41^ and aerobic fitness in both childhood and adulthood^41–43^. Other studies have found that both very low and very high birth weights are associated with a lower likelihood of engaging in leisure-time physical activity^44^. A prospective cohort study from Brazil found that higher birth weight predicted lower physical activity and more sedentary time in women, but these findings may have been confounded by adult obesity^45^. However, other studies found no associations between birth weight and later-life physical exercise levels, e.g. in children whose physical activity was objectively monitored^46,47^.

In addition, adolescents and adults who were born with low or extremely low birth weight reported lower levels of physical activity or participation in sports activities compared to their normal birth weight peers^48,49^. These results suggest that an adverse prenatal environment may program either the physiological capacity or behavioral willingness to engage in voluntary physical activity in adulthood^50^. One possible mechanism underlying reduced physiological capacity for spontaneous physical activity in individuals with lower birth weight could involve alterations in skeletal muscle morphology, such as changes in muscle fiber composition and fiber size, that impair physical and metabolic muscle performance^51^.

An alternative mechanism may involve the development of adverse mitochondrial phenotypes in the context of intrauterine growth restrictions (IUGR). For instance, it has been shown that placental tissue from IUGR pregnancies exhibits a significant reduction in the enzymatic activity of mitochondrial Complex I, accompanied by decreased citrate synthase activity in neonatal cord blood mononuclear cells^52^. This mitochondrial imbalance may contribute to secondary cardiovascular remodeling in infants and bioenergetic deficits leading to decreased capacity for physical activity in the adulthood. It has also been shown that the cardiorespiratory effectiveness could be impaired among individuals with early developmental growth restriction trajectories, resulting from lower lung function (e.g. lower diffusing capacity) or reduced cardiac structure and output^53^.

On the other hand, mechanisms related to behavioral willingness may involve alterations in dopaminergic signaling, which can reduce motivation for physical activity and contribute to greater fatigue perception^54^. For instance, a recent animal study demonstrated that introducing dopaminergic blockade in rats was associated with more rapid hypothalamic activation and a marked reduction in exercise time until fatigue - from approximately 59 min in control animals to just 13 min in the test group^54^. Given dopamine’s well-established role in reward processing, mood regulation and motor control, changes in dopaminergic signaling may also lead to diminished incentive to be physically active due to a weakened sense of gratification following physical activity^55,56^.

In this study, we analyzed relationships between the size at birth (birth weight and ponderal index) and daily levels of physical activity during three full menstrual cycles in adult women. Further, we analyzed relationships between size at birth and time spent in light, moderate and vigorous physical activity. Although birth weight is commonly used as an indicator of fetal conditions, the ponderal index, which accounts for body proportions, may provide additional insights into fetal growth restriction^57,58^.

We hypothesized that individuals born with smaller size (i.e., birth weight and ponderal index) are less physically active and more reluctant to increase physical activity (i.e., being more energy-thrifty) than adults born with larger size. Additionally, we hypothesized that energy should be conserved particularly during high-intensity recreational physical activity.

## Materials and methods

### Study participants and procedure

A total of 234 women from Krakow, Poland participated in the research project on physical activity, sex hormones and size at birth^59^. Data on birth size were available for 185 participants. The final study sample included 136 women (mean age 26.6; SD 4.24). Women were excluded from the statistical analyses if they did not provide reliable accelerometer data, either due to failure to wear the device or incorrect readings. Additionally, one participant was excluded due to a suspected error in the birth measurements recorded in her health record, and another was excluded for exhibiting exceptionally high levels of physical activity across all three menstrual cycles. Participants were recruited between October 2019 and October 2023 through local media advertisements, social media posts, and promotional campaigns. Initially, a telephone interview was conducted to verify that each participant met the study inclusion criteria related to e.g., age, body size, health status, and menstrual cycles. Women who met the eligibility criteria were invited to attend in-person meeting. During the meeting, a trained research assistant conducted an interview with each participant. The interview included a general questionnaire covering topics such as demographic information (e.g., age, education measured in completed years of schooling), lifestyle factors, overall health, and reproductive health. Additionally, body height, weight, and fat percentage were measured by a trained team member. Body weight and body fat percentage were assessed using a bioimpedance TANITA Body Composition Monitor (Tanita BC-545 N). Eligible women were between 20 and 36 years old, reported regular menstrual cycles (with cycle length fluctuations of no more than 5 days), and had a Body Mass Index (BMI) within the normal range (18.5 to 24.9 kg/m^2^). Participants were required to have no medical contraindications to engage in intense physical activity, as confirmed by a physician from the research team. The study complied with the ethical standards specified in the Declaration of Helsinki and was approved by the Bioethics Committee of the Jagiellonian University (approval number: 1072.6120.47.2018). Written informed consent was obtained from all participants.

### Birth parameters

Birth-related parameters, namely birth weight, body length and gestational week at delivery, were extracted from the participants’ personal child health records. In Poland, child health booklets are issued by the hospital after the child’s birth, based on the regulation issued by the Polish Minister of Health in 1959^60^. Booklets containing information on the child’s birth characteristics (e.g., a length of pregnancy, type of delivery, size at birth, Apgar score), were used by medical personnel to record subsequent health condition, diseases and vaccinations of a child and were kept by parents at home. For each participant the ponderal index (also known as the corpulence index or Rohrer’s Index - an indicator of newborn leanness)^61^ was calculated by dividing birth weight (kg) by cubed body length (m^3^).

### Physical activity

Participants’ physical activity was monitored over three consecutive menstrual cycles. Women were requested to keep their usual level of activity for the first cycle and increase their physical activity starting from the beginning of cycle 2 and retain or further increase activity in cycle 3. Each woman was provided with a pass to a large chain of gyms and fitness clubs for unlimited access to training sessions and was instructed to engage in at least 180 min of moderate to vigorous physical activity per week, choosing activities according to her preference. This target was based on the World Health Organization (WHO) guidelines, which recommend that adults engage in at least 150 min of moderate-intensity or 75 min of vigorous-intensity activity per week to gain substantial health benefits^7^. To track their physical activity participants were given a Fitbit Alta HR wristband accelerometer (Fitbit, Inc.; San Francisco, CA, USA) and asked to wear it continuously for all three menstrual cycles, except during showers, baths, or swimming. Multiple studies have confirmed relatively high accuracy of Fitbit devices in estimating steps and heart rate^62,63^.

Additionally, as part of the study protocol, participants were asked to record each session of physical activity (type and duration) in an online calendar shared with the research team. This recording was intended not only as an additional source of data but also as a behavioral prompt, reinforcing self-awareness and potentially motivating adherence to the physical activity recommendations. The research team had real-time access to the entries, though no feedback was provided to participants during the study.

To further support physical activity adherence, the Fitbit devices were programmed to send inactivity reminders during cycles 2 and 3. These notifications prompted participants to engage in physical activity after prolonged periods of sedentary behavior, encouraging them to meet their activity goals. These reminders were designed to offer a gentle nudge to encourage exercise.

The estimates of daily steps, total energy expenditure and active minutes from the Fitbit devices were obtained directly from the participants’ individual accounts on the Fitbit platform. The Fitbit device recorded daily physical activity into pre-set categories using metabolic equivalents (METs). The data was downloaded as daily step counts, and minutes spent lightly active (intensity < 3 METs), minutes spent fairly active (3–6 METs), and minutes spent very active (> 6 METs). Total energy expenditure was calculated by the Fitbit device as the combined total of basal metabolic rate (BMR) and energy expended on all activities. Fitbit physical activity data was available for an average of 92% of days in cycle 1, 94% in cycle 2, and 90% in cycle 3. We considered accelerometer readings valid if they indicated at least 500 steps per day and the device was worn for at least 7 days during a given menstrual cycle. According to the literature, data from 4 days of accelerometer wear, when there are no restrictions on consecutive measurement days, and five days of consecutive wear, provide a valid estimate of physical activity over the course of a month^64^. We calculated the average daily step count, and the average time spent in light, moderate, and vigorous activity (in minutes) for each day of the cycle during each menstrual cycle.

### Statistical analyzes

The relationship between birth weight and physical activity levels across the three menstrual cycles was analyzed using multiple regression. Physical activity was included in subsequent models either as step count, total energy expenditure or average number of minutes per day spent in light, moderate or vigorous physical activities calculated separately for each of three of consecutive menstrual cycles. To control for potential confounders, we included age, education, and gestational age at birth in the model with total energy expenditure as the dependent variable, and additionally controlled for body fat percentage in models analyzing step count and time spent in light, moderate, and vigorous physical activity. All analyses were repeated with the ponderal index, instead of birth weight, as the independent variable. To control for the risk of type I error due to multiple comparisons, the Benjamini-Hochberg procedure was applied to adjust p-values, controlling the false discovery rate (FDR). Adjusted p-values were considered statistically significant if the FDR was < 0.05. The data were analyzed using the IBM SPSS Statistics 29.0.0.0 version for Mac OS package and StatSoft Statistica 13.1 PL statistical package.

## Results

The descriptive characteristics of the study group are presented in Table 1. Data on physical activity are presented in Table 2. The number of observations for individual variables may vary slightly due to missing data or participants’ withdrawal from the study. The level of physical activity, reflected in the average daily step count and the time spent in moderate and vigorous physical activity, in cycles 2 and 3 was significantly higher compared to cycle 1 (Table 2).

**Table 1:** Characteristics of the study group.

Variable	*N*	Mean	SD	Min	Max
Age (years)	136	26.6	4.24	20.0	36.0
Education (completed years of schooling)	136	16.6	2.93	12.0	30.0
Birth weight (g)	136	3304.7	548.13	1400.0	4600.0
Birth length (cm)	136	54.2	3.27	45.0	66.0
Ponderal index (kg/m^3^)	136	20.7	2.42	14.4	30.2
Gestational age at birth (weeks)	136	39.4	1.81	31.0	44.0
Adult body fat (%)	136	25.7	6.55	13.9	43.4

**Table 2:** Physical activity levels across three menstrual cycles (accelerometers outcomes).

Accelerometer outcomes	Cycle 1			Cycle 2			Cycle 3			Repeated measures ANOVA	
	*n*	Mean (SD)	Min; Max	*n*	Mean (SD)	Min; Max	*n*	Mean (SD)	Min; Max	F	*p*
Steps per day	136	9231.6 (2627.06)	1961.4; 16769.9	132	10453.8 (2770.35)	4191.9; 18116.7	129	10065.9 (2613.75)	3335.7; 19683.0	24.93	< 0.001
Total energy expenditure (kcal/day)	136	2104.8 (258.58)	1605.9; 2846.5	132	2185.6 (293.92)	1593.7; 3210.6	129	2162.1 (272.82)	1514.6; 3084.4	23.91	< 0.001
Total light physical activity (minutes/day)	136	239.2 (64.3)	7.9; 404.3	130	243.6 (63.23)	10.2; 420.8	128	240.2 (61.49)	94.9; 476.3	1.29	0.276
Total moderate physical activity (minutes/day)	136	19.5 (13.3)	0.0; 85.7	130	25.8 (16.93)	0.1; 131.5	128	25.0 (13.56)	1.3; 66.9	23.35	< 0.001
Total vigorous physical activity (minutes/day)	136	16.0 (10.46)	0.0; 52.9	130	24.8 (15.89)	0.1; 82.1	128	22.8 (13.88)	1.0; 65.4	48.70	< 0.001

The ponderal index was positively related to duration of vigorous physical activity across all menstrual cycles - both in the cycle 1 with the usual physical activity (*p* < 0.001) and in the two cycles with increased physical activity (cycle 2: *p* = 0.011; cycle 3: *p* = 0.004), when controlled for participants age, education, body fat percentage and gestational age at birth (Table 3). We also observed a positive relationship between birth weight and total energy expenditure in all menstrual cycles (cycle 1: *p* = 0.014; cycle 2: *p* = 0.008; cycle 3: *p* = 0.016). These associations remained statistically significant after applying the Benjamini-Hochberg correction for multiple comparisons (FDR < 0.05).

No statistically significant relationships were observed between light or moderate physical activity levels and either birth weight or ponderal index. Additionally, the average number of steps taken per day was not significantly associated with birth weight or ponderal index (Table 3).

**Table 3:** Relationships between birth weight and ponderal index with physical activity in women. Results from multiple regression models.

		Cycle 1			Cycle 2			Cycle 3		
		β	SE	*p*	β	SE	*p*	β	SE	*p*
Total energy expenditure * (kcal/day)										
Birth weight (g)		0.11	0.05	**0.014** **0.016†**	0.14	0.05	**0.008** **0.016†**	0.12	0.05	**0.016** **0.016†**
Ponderal index (kg/m^3^)		13.16	9.29	0.159	9.54	10.66	0.373	9.90	9.89	0.319
Steps per day **										
Birth weight (g)		-0.13	0.48	0.195	-0.10	0.51	0.308	-0.10	0.48	0.326
Ponderal index (kg/m^3^)		0.05	93.75	0.59	0.04	98.82	0.637	0.04	93.09	0.643
Total light physical activity ** (minutes/day)										
Birth weight (g)		-0.10	0.01	0.309	-0.01	0.01	0.967	-0.16	0.11	0.134
Ponderal index (kg/m^3^)		-0.13	2.29	0.127	-0.14	2.21	0.105	-0.15	2.15	0.081
Total moderate physical activity ** (minutes/day)										
Birth weight (g)		-0.06	0.00	0.575	-0.11	0.00	0.309	0.02	0.00	0.878
Ponderal index (kg/m^3^)		0.08	0.48	0.356	0.03	0.61	0.744	0.14	0.48	0.130
Total vigorous physical activity ** (minutes/day)										
Birth weight (g)		0.08	0.00	0.456	0.03	0.00	0.808	0.04	0.00	0.670
Ponderal index (kg/m^3^)		0.28	0.36	**< 0.001** **0.003†**	0.22	0.54	**0.011** **0.011†**	0.25	0.47	**0.004** **0.006†**

* Note: Model adjusted for age, education and gestational age at birth.

** Note: Models adjusted for age, education body fat and gestational age at birth.

† Benjamini-Hochberg Adjusted *P* value.

Significant values are in bold.

## Discussion

In healthy, adult women of reproductive age the quality of fetal developmental conditions may be associated with physical activity in later life. Women developing in energy-scarce conditions, as indicated by their smaller size at birth, had lower levels of total energy expenditure and were less likely to engage in vigorous physical activity. This suggests that poor developmental conditions may “program” individual to be energetically efficient not only via physiological mechanisms leading to saving energy e.g., by permanent changes in glucose-insulin metabolism^12^ but also via behavioral mechanisms leading to lower physical activity, thus saving energy by expending less of it.

One potential behavioral mechanism involves alterations in the development of dopamine signaling, which plays a well-established role in the brain’s reward system and the voluntary control of movement^65^. This makes it a strong candidate for investigation in the context of motivation for spontaneous physical activity^66^. Numerous animal studies have shown that dopamine levels are closely linked to motivation for spontaneous activity, such as wheel running, with more evidence pointing toward reward system, rather than motor control, as the likely explanation^66^.

Importantly, the development of dopaminergic signaling, shaping brain function at molecular, circuit, and behavioral levels, may be particularly sensitive to early-life conditions and may exert lasting effects into adulthood^65,67^. Future research should investigate how specific dopamine signaling cascades that mediate the rewarding aspects of physical activity develop, and how early-life influences may shape these mechanisms.

While size at birth was related to total energy expenditure and vigorous physical activity, women born smaller did not differ from women with larger birth size in time spent in low and moderate physical activity. This may stem from the fact that some activities such as foraging, walking, food preparation and childcare were essential for survival for our evolutionary ancestors and could not be avoided, regardless of early developmental factors. However, energy expenditure can be reduced by avoiding vigorous physical activity, as we observed in studied women across all three menstrual cycles. These findings align with Pontzer’s constrained energy expenditure model^68^ which suggests that total energy expenditure is regulated, and that increased energy spent on particular activities may be compensated by reductions in other areas to maintain the overall energy balance. There are different predictions concerning spending energy in activities with high intensity which are energetically costly. Namely, between the ability to engage in high intensity activities in life-threatening situations (e.g. escaping a predator) versus in expending energy in high intensity activities that are not necessary for survival (e.g. leisure type activities, exercise). In individuals with small size at birth, the former should be preserved, but not necessary the latter.

Several strengths of our study should be noted. First, our study participants represented wide range of birth weights (1400–4600 g), which adds variability and reflects real-world differences^69^. Physical activity was objectively measured using accelerometers, following standardized protocols to maintain consistency. We assessed physical activity based on at least seven days per month of proper accelerometer use, which is widely regarded as a reliable method for capturing habitual activity^64^. Physical activity was measured for about three months (i.e., three menstrual cycles) and included a cycle with lower activity and two cycles during which women were requested to increase their activity. While hormonal fluctuations across the menstrual cycle (particularly changes in estrogen and progesterone) can affect both exercise capacity and behavioral motivation for physical activity, current evidence suggests that these effects are relatively modest and vary considerably between individuals^70^. Our within-subject design allowed us to capture natural hormonal fluctuations without synchronizing data collection to specific menstrual cycle phases. By observing each participant across three complete cycles, we accounted for cycle-related variability on an individual level. This approach enhances internal validity and strengthens the reliability of findings, particularly in analyses related to menstrual cycle effects.

In this study women were not only encouraged to be physically active but also received passes to fitness clubs which provided an opportunity to increase and to maintain high physical activity. Furthermore, early developmental markers such as birth weight and birth length were extracted directly from medical records, ensuring accuracy and reducing recall bias, as these measurements were performed and written down at time of birth by trained medical personnel. Validation studies have shown that medically documented birth data such as birth weight and gestational age are generally accurate and reliable for epidemiological research^71,72^. Finally, while birth weight is commonly used as an indicator of fetal conditions, we also tested the relationships with the ponderal index, which accounts for body proportions and corpulence at birth, and may provide additional insights into fetal growth and maternal nutritional status^57,61,73–76^. To the best of our knowledge, our study is the first to combine (i) long-term accelerometer-based monitoring of physical activity across three consecutive menstrual cycles, (ii) a within-subject design that includes both habitual and instructed activity phases, and (iii) dual indicators of birth size (birth weight and ponderal index). This design allows for a more reliable analysis of how early-life growth conditions may influence physical activity behavior in adulthood.

Some study limitations should be taken into account. Our study sample consisted solely of young, healthy women of selected age and BMI ranges. These results may not be replicable in different groups of women, e.g. older, with poorer heath status or overweight, mostly due to confounding effects that these factors may have on physical activity. In particular, we included only participants within the normal BMI range to minimize the metabolic variability that could arise from underweight, overweight or obesity. Birth weight is associated with long-term alterations in metabolic pathways, including impaired glucose and insulin metabolism, insulin resistance, dysregulation of carbohydrate and lipid profiles, and hormonal axis disturbances such as altered cortisol secretion^77–81^. These processes may influence both energy availability and motivation or capacity for physical activity^51,82–85^. Including women with BMI outside of normal values could have introduced additional metabolic confounders, making it difficult to isolate the relationship between birth weight and physical activity. However, this choice also limits the generalizability of our findings to women with overweight or obesity, for whom both metabolic and behavioral patterns may differ. Some constrains also could be attributed to physical activity measurements. Fitbit wristband accelerometers were recently shown to be error prone^63^. The accuracy of the data depends on proper device use, which can vary between participants and affect reliability of measurements. While previous studies have shown that Fitbit devices reliably estimate the number of steps and energy expenditure during basic physical activities such as walking and running^86^ they may not accurately capture activities like cycling and cannot be worn while swimming, potentially leading to underestimation of the activity. Additionally, the Fitbit devices classify physical activity intensity levels reasonably well compared to research-grade accelerometers^62,87^ but their accuracy in estimating energy expenditure is limited and can vary depending on the specific context^62^. Furthermore, our study did not account for potential confounding factors such as childhood socioeconomic status, early-life physical activity habits, or parental influences. Parental and caregiving responsibilities may also affect physical activity levels. Although we did not directly assess these responsibilities, a comparison between women with (*n* = 10, 7% of the total sample) and without children revealed no significant differences in time spent in vigorous activity, total energy expenditure, or average daily step count (p-value ranged from 0.102 to 0.977), suggesting that, within our sample, having children did not substantially impact physical activity. Nevertheless, we acknowledge that caregiving duties could influence leisure time physical activity in other populations. These limitations highlight the need for future research, particularly studies involving more diverse populations, additional potential confounders (i.e. socioeconomic of family situation) and those that complement accelerometer data with additional methods of physical activity assessment, such as the doubly labelled water technique^88^.

In conclusion, our results contribute to better understanding how early life conditions influence health and disease, following the Barker’s hypothesis, which underpins the Developmental Origins of Health and Disease (DOHaD) framework. This paradigm highlights the critical role of developmental plasticity in shaping long-term health outcomes^18,89^. This plasticity allows the body to adapt to varying environmental post-natal conditions. Early life experiences, such as fetal deficiency of energetic resources or even malnutrition, significantly influence the risk of developing chronic diseases later in life^20,21,24^. Furthermore, insufficient physical activity remains a major contributor to the development of these chronic conditions. Our results support the hypothesis that adult women with smaller birth size tend to be less physically active and more resistant to increasing their physical activity levels compared to women with larger birth size. Energy is conserved by avoiding vigorous physical activity, even when participating in a project designed to boost physical activity to a moderate-to-vigorous level. Although the observed effect size was modest (β = 0.28), it corresponds to approximately 17 additional seconds of vigorous activity per day for unit increase in ponderal index, which may accumulate to meaningful health benefits over time. Previous research has shown that even small amounts of moderate-to-vigorous physical activity (MVPA) can yield significant health benefits. For example, Hupin et al. found that just 15 min of MVPA per day is associated with a 22% reduction in mortality risk among older adults^90^. Similarly, Wen et al. demonstrated that 92 min of physical activity per week can increase life expectancy by three years and reduce mortality by 14%, emphasizing the public health importance of promoting physical activity, particularly among individuals with early developmental vulnerabilities^90,91^. While we acknowledge that other factors, including childhood socioeconomic status, early-life physical activity and parental behavior, may influence adult activity levels, our findings offer important insights into how early-life conditions shape long-term physical activity patterns and disease susceptibility, highlighting the value of early interventions and the promotion of physical activity as strategies to mitigate future health risks. Since early developmental conditions play a key role in programming the risk of chronic diseases in adulthood, particularly those for which high levels of physical activity are risk reducing factors, we suggest that body size at birth may be an important factor to consider in studies attempting to understand barriers to engage in regular physical activity. While public health guidelines remain universal, individuals born with lower birth weight may need additional support or tailored interventions to meet physical activity recommendations, given their greater likelihood of reduced activity levels. Lower activity levels in this group may stem not only from behavioral tendencies but also physiological constraints like reduced muscle mass or aerobic capacity, along with metabolic, psychological, environmental, and genetic factors, highlighting the complexity of influences on physical activity and the need for multifaceted approach in future research. The difficulty in distinguishing whether lower physical activity in individuals with adverse prenatal environments results from physiological capacity or behavioral factors has important implications^92,93^. The lack of clear differentiation between these mechanisms makes the design of effective interventions challenging. If reduced physical activity primarily stems from physiological limitations, interventions should prioritize enhancing exercise capacity (e.g. through targeted, regular training). However, if altered behavior is the primary factor educational initiatives and motivational support will be essential.

## Data Availability

The datasets used and analyzed during the current study are available from the corresponding author on a reasonable request.
